# Understanding patient outcomes to develop a multimorbidity adapted patient-reported outcomes measure: a qualitative description of patient and provider perspectives

**DOI:** 10.1186/s12955-021-01689-w

**Published:** 2021-02-04

**Authors:** Maxime Sasseville, Maud-Christine Chouinard, Martin Fortin

**Affiliations:** 1 Department of Health Sciences, Université du Québec à Chicoutimi, 555 boulebard université, Chicoutimi, Québec G7H 2B1 Canada; 2 Nursing Faculty, Université Laval, 2325, rue de l’Université, Québec, QC G1V 0A6 Canada; 3 Nursing Faculty, Université de Montréal, Pavillon Marguerite-d’Youville, C.P. 6128 succ. Centre-ville, Montréal, QC H3C 3J7 Canada; 4 Research Chair on Chronic Diseases in Primary Care, Department of Family and Emergency Medicine, Université de Sherbrooke, 3001, 12e Avenue Nord, Sherbrooke, QC J1H 5N4 Canada

**Keywords:** Chronic diseases, Qualitative description, Multimorbidity, Patient-centred care, Patient-reported outcomes, Self-management

## Abstract

**Background:**

Multimorbidity is a complex health situation that requires interventions tailored to patient needs; the outcomes of such interventions are difficult to evaluate. The purpose of this study was to describe the outcomes of patient-centred interventions for people with multimorbidity from the patients’ and healthcare providers’ perspectives.

**Methods:**

This study followed a qualitative descriptive design. Nine patients with multimorbidity and 18 healthcare professionals (nurses, general practitioners, nutritionists, and physical and respiratory therapists), participating in a multimorbidity-adapted intervention in primary care were recruited. Data were collected using semi-structured interviews with 12 open-ended questions. Triangulation of disciplines among interviewers, research team debriefing, data saturation assessment and iterative data collection and analysis ensured a rigorous research process.

**Results:**

Outcome constructs described by participants covered a wide range of themes and were grouped into seven outcome domains: Health Management, Physical Health, Functional Status, Psychosocial Health, Health-related Behaviours, General Health and Health Services. The description of constructs by stakeholders provides valuable insight on how outcomes are experienced and worded by patients.

**Conclusion:**

Participants described a wide range of outcome constructs, which were relevant to and observable by patients and were in line with the clinical reality. The description provides a portrait of multimorbidity-adapted intervention outcomes that are significant for the selection and development of clinical research outcome measures.

## Introduction/background

Multimorbidity is the co-occurrence of multiple chronic conditions in the same individual [1] and is associated with poorer quality of life [2, 3], psychological distress [4–6], lower physical function [7], polypharmacy and adverse drug events [8] and care duplicity and inconsistencies [9, 10]. Chronic disease management interventions adapted for persons with multimorbidity are used as a patient-centred method of care. However, clear evidence of their effectiveness is still lacking, partly because existing measures are not adapted to these interventions due to their multidimensional nature [1].

Patient-centred care is defined as the individualized provision of care using a compassionate approach and incorporating contextual elements to support patient self-determination [11]. Thus, multimorbidity-adapted intervention based on patient-centred care focuses on encouraging patient self-management and decision-making and the individualization of patient care [10, 11]. A recently updated systematic review on these interventions identified the need for an outcome measure adapted to multimorbidity [1], as most generic measures do not cover the full array of effects identified in the literature [12].

The direct report of a health condition by a patient without a clinician’s interpretation is called a patient-reported outcome measurement (PROM) and may be in line with the patient-centred approach of interventions adapted to multimorbidity [13, 14]. The Canadian Institute for Health Information point that PROM are essential to understand if interventions are influencing the patient quality of life [15]. PROM is also supported by the health ministers of the member countries in the Organisation for Economic Co-operation and Development (OECD), who have identified its value in measuring what matters to patients [16].

In keeping with the recommendations for minimum standards for PROM, the International Society for Quality of Life Research (ISOQOL) identified that a PROM should be based on a measurement model developed using the intended population [17]. Guidelines for developing a PROM suggests that qualitative input is necessary to develop the domains, constructs and items to be measured to improve content validity [13, 18]. For PROM development, the aim of the sampling is to obtain representative experiences not representative population, thus multiple subgroups may be used [19]. Given that patient outcomes of a clinical intervention can be observed by both patients and healthcare providers, both subgroups were considered [20]. As yet, there is still no qualitative understanding of which outcomes result from a patient-centred multimorbidity intervention.

The main goal of this qualitative study was to describe patient and healthcare providers' perspectives on patient outcomes resulting from a patient-centred care intervention for patients with multimorbidity. The analysis also wanted to inform the development of a PROM for chronic disease management interventions for persons with multimorbidity. As a first step of the development of a PROM, a scoping review was completed on PROM used in multimorbidity-adapted interventions [21].

## Method

The study used a qualitative descriptive design [20] and *the Consolidated criteria for reporting qualitative research (COREQ) were used to guide, conduct and report this study* [22]*.*

### Recruitment and sample

Participants were recruited after they participated in an interdisciplinary chronic disease management intervention conducted in Québec, Canada and clinically implemented in six family medicine groups (FMGs). This intervention was based on the patient-centered care orientations set out by the Québec government’s department of health concerning the management of people with chronic conditions, i.e., self-management support, patient-centred care, motivational interviewing, interprofessional collaboration and integration of services [23]. In the participating FMGs, healthcare professionals (nutritionists, kinesiologists and other professionals) were added to primary care teams of physicians and nurses to work collaboratively in providing care for patients with multiples chronic diseases and risk factors [24]. The participating professionals received an average of 7.8 h of training on patient-centered care self-management support and interprofessional collaboration prior to implementation in FMGs. Patients with three or more chronic diseases received a patient-centred chronic disease management intervention adapted to their needs and health objectives by the interdisciplinary primary care team [24]. Patients received in average 2.6 h of interventions over the 4-month period of the intervention study.

The sample in this study includes patients and healthcare providers. We used a maximum variation sampling method based on chronic conditions, gender and age to recruit the patients sample from the intervention [19]. The healthcare providers recruited for the study participated in the intervention and were interviewed to expand the description of patients’ outcome experience.

### Data collection

We used semi-structured interviews using two interview guides, i.e., one for each group, the only difference being in the wording of the questions, in order to focus on patient outcomes in the interviews with the providers. The structure of the guides was based on the outcome domains identified in the aforementioned scoping review [21]. The questions in the preliminary interview guide used outcomes constructs identified in MM interventions studies grouped in domains: health management, functional status, psychosocial health, health-related behaviours, general health and health services. The last question was formulated in general to allow patients to express if the intervention helped them to change other aspects of their life. Table 1 provides the questions asked during the patient interviews. The interview guide also included sub-questions to clarify or deepen the description. For example, the question “To what extent has the intervention influenced your physical health?” could be followed by “Which symptoms were influenced?” or “Could you give precisions on how much [symptom] was influenced?”. The interview guides were pretested with each group of participants (patients and healthcare providers) and subsequently minor wording changes were made [25]. Individual interviews were conducted in person or by phone (one interview) by five interviewers from different disciplines (nursing, medicine and social sciences) over an 8-month period in 2016–2017. Interviews were audio-recorded, transcribed verbatim and then imported into NVivo 11 software for data sorting and analysis. Field notes were taken to report the general feeling or specific observations made during the interview.

**Table 1:** Interview questions from the adjusted version of the guide

For which chronic conditions do you get support from healthcare providers in the intervention?
How are you managing your health at home?
To what extent has the intervention influenced your health status?
To what extent has the intervention influenced your knowledge about your health?
To what extent has the intervention influenced your physical health?
To what extent has the intervention influenced your quality of life?
To what extent has the intervention influenced your mood?
To what extent has the intervention influenced your ability to have health-related behaviours?
To what extent has the intervention influenced your ability to do social activities with family and friends?
To what extent has the intervention influenced your ability to get support from family and friends?
To what extent has the intervention helped you to attain your health objectives?
To what extent has the intervention helped you to change other parts of your life?

### Data analysis

Data was collected and analyzed iteratively to obtain an evolving thematic analysis and the research team’s clinical experience was used to generate themes relevant to a clinical context [25]. In line with the Thorne methodology [26], the previously conducted scoping review on PROM used in MM studies was used as a preliminary framework to create the initial coding scheme. The first author (MS) conducted the coding and evolving thematic analysis and MF and MCC validated the process in biweekly meetings using representative quotes. Data saturation was documented by subgroups of participants using a data saturation table [25].

## Results

Twenty-seven participants were recruited across all six settings. The sample consisted of nine patients with multimorbidity, nine nurses, two physicians, four nutritionists, two physical therapists and one respiratory therapist. Participant characteristics are presented in Table 2. Interview length varied from 26 to 71 min and data saturation was reached after interviewing seven patients and 13 providers; two more patients and one of each type of provider [5] were interviewed with no new themes emerging.

**Table 2:** Sample characteristics

Patient characteristics (n = 9)	
Sex (male), n (%)	5 (55.6)
Age (mean, in years)	55
Education level, n (%)	
High school	1 (11.1)
College	6 (66.7)
University	2 (22.2)
Marital status, n (%)	
Married	6 (66.7)
Divorced or separated	3 (33.3)
Single never married	0 (0)
Working, n (%)	1 (11.1)
Number of chronic conditions, n (%)	
3–4	4 (44.4)
5–6	3 (33.3)
7 or more	2 (22.2)

Healthcare providers (n = 18)	
Profession, n (%)	
Nurses	9 (50)
Physicians	2 (11.1)
Nutritionists	4 (22.2)
Physical therapists	2 (11.1)
Respiratory therapist	1 (5.6)
Age group (in years), n (%)	
Under 30	5 (27.8)
30–39	5 (27.8)
40–49	5 (27.8)
50–59	1 (5.6)
60 and older	2 (11.1)
Length of practice (in years), n (%)	
Fewer than 5	4 (22.2)
6–10	2 (11.1)
11–15	6 (33.3)
16–20	2 (11.1)
21–25	2 (11.1)
More than 25	2 (11.1)

Participants described 19 outcomes that we grouped according to the domain classification from our previous scoping review [21]: Health Management, Physical Health, Functional Status, Psychosocial Health, Health-related Behaviours, General Health and Health Services. Thematic organization of domains and outcomes that have emerged from the qualitative description (in comparison with the preliminary framework) are presented in Table 3.

**Table 3:** Organization of outcomes

Domain	Outcomes
Health management	Awareness^a^
	Knowledge acquisition
	Power over health
	Self-efficacy
	Health goal attainment
	Self-management
Physical health^a^	Pain and physical symptoms^a^
	Energy^a^
	Weight control^a^
Functional Status	Autonomy in daily activities
Psychosocial health	Anxiety
	Well-being
Health-related behaviours	Physical activity
	Healthy eating
	Smoking habits^a^
	Alcohol consumption habits^a^
General health	Quality of life
Health services	Patient satisfaction^a^
	Services use

^a^Emerged from the qualitative study (compared with preliminary framework)

### Health management domain

Talking about their conditions and health behaviours helped patients by making them more aware of how they value their health and realize that their health is important to them.

Confessions about alcohol consumption and smoking behaviours raised my awareness about the state of my health state [pause]. It made me aware that health was important for me. (patient 1)

Knowledge was acquired concerning health-related behaviours, symptom management and long-term complications. A participant explained that he now knows how to adopt healthy eating behaviours using examples of plate size to demonstrate his understanding. Participants described knowledge acquisition as being newly mindful of the long-term complication of chronic diseases that prompted to better manage their health condition.

I now have some tricks for doing physical activity without getting a “hypo” [hypoglycemia]. (patient 4)

Participants identified a new awareness of the power they had over their health and how it helped them regain control over it. In relation to power over health, a process of transfer of power over health decisions was described by participants.

I have a healthcare team, but part of the power is mine, I want to take back the power over my life. (patient 7).

Participants described feeling an increased capacity to self-manage their health and knowing when external assistance is necessary. Self-efficacy was further described as a patient’s initial success, producing a feeling of capacity. Being able to express, pursue and attain their own health goals was an important outcome for the participants.

They are proud, it gives them so much self-confidence, to be able to have successes. It is wonderful, it gives them a feeling of capacity. (provider 3)

Moreover, participants mentioned that goal attainment should be the principal outcome pursued by an intervention for patients with multimorbidity.

Interviewer: Your objective was to not take medication? Participant: Yes, if I follow the recommendations for my eating habits, I should be able to obtain an acceptable cholesterol level. (patient 9)

Participants described self-management of health conditions as an overarching result of other outcomes. Learning about their health conditions, complications and health-related behaviours were key components identified as helping patients act to manage their conditions.

[…] they [patients] are going to better understand their diseases, and then feel responsible for their management. (provider 12)

### Physical health domain

Physical health outcomes were described as an improvement in multiple physical manifestations of chronic conditions, including pain improvable by the multimorbidity intervention.

I have less pain, better digestion, less diarrhea, less stomach pain. (patient 7)

Increased energy levels to complete daily tasks and health-related behaviours were reported, helping patients to “get moving.” Participants added that improved disease management leads to an increase in available energy for daily tasks.

It gives me fuel to start the day, it’s getting me moving. (patient 7)

Weight loss and the ability to prevent further weight gain also constituted a relevant outcome described by participants. Regarding weight control, participants pointed out that a stable weight should also be considered significant for some patients.

I have lost 15 pounds since July, and I am maintaining it. (patient 5)

### Functional status domain

Overall physical health significantly influenced autonomy in daily activities. Participants further stated that health status improvement and pain control had an impact on patient autonomy in daily activities such as cooking, cleaning and getting dressed. It was described that the intervention helped patients understand the interlinked nature of their condition, indicating that improvements in health status increased their ability to complete daily tasks.

They felt less limited, with fewer physical, psychological and physiological limitations that affected their daily living. (provider 10)

### Psychosocial health domain

Participants reported that engaging in self-management reduced their anxiety. It was also identified that knowledge acquisition improved specific stressors and further acknowledged that transferring control of the health situation to patients was beneficial for patient anxiety.

It helped me get moving and activate my brain, and that also calmed my anxiety. (patient 7)

Well-being was described as a process of living with a positive mindset and accepting one’s health situation. Participants reported guilt reduction when they were told that they could make mistakes while engaging in their process of change.

It helped me to live better, to better accept [pause] and live my life in relation with my health status. (patient 1)

### Health-related behaviours domain

The physical activity outcome was described as new activities or changes in the type and duration of physical activities reported by patients.

I have more energy and I want to get back into it [physical activity]. (patient 8)

Several outcomes were reported regarding patients’ eating behaviours including portion size, meal schedule, choice of food and the introduction of fruits and vegetables.

But that’s it, it’s the quantity [of food] that I need to be aware of at home. (patient 08)

It’s about what is good to eat, choosing fish for example, and avoiding bad food, like high-fat food. (patient 9)

Participants reported some improvement in smoking habits by cutting down from daily smoking to occasional smoking.

My family physician told me several times that I needed to stop smoking and prescribed [nicotine] patches, but I was always relapsing, but the intervention was what I needed [to stop]. (patient 10).

A decrease in alcohol consumption was described as a change to non-alcoholic alternatives or fewer alcoholic beverages per day. Participants further explained that a reduction in alcohol consumption was linked to patient-centred objectives and readiness to change.

Patients tell me: Since I’ve been drinking more water I have reduced my alcohol consumption. (provider 3).

### General health domain

Reports of improved quality of life outcomes were described by participants as an overall effect of all factors influencing health. In terms of a general health outcome, participants also reported feeling healthier than before, saying that this had a positive influence on their overall mood. Participants described a life-changing experience brought by changing health habits and way of seeing health.

With all the services, I would say that I have an 80% increase in quality of life […] you know, from having a place to be heard, get moral support and orientation. (patient 7).

### Health services domain

Patients stated that their satisfaction with the health services received was closely related to a feeling of safety and an appropriate follow-up.

I feel like I am in good hands, when I come here I feel like I’m going to get the answers that I rightfully deserve, I feel safe. (patient 4).

Primary care physicians reported seeing patients less frequently because they were being followed up by an entire team of healthcare providers.

I saw these patients less, I kind of lost sight of them because they went to improve their condition with other people [healthcare providers]. (provider 1)

## Discussion

This study sought to describe patients’ and healthcare providers' perspectives on patient outcomes resulting from a patient-centred care intervention for individuals with multimorbidity.

Patients and providers described health management outcomes as processes and acquired skills supporting self-management for people with multimorbidity. The description of the Health Management outcome domain includes awareness, knowledge acquisition, power over health, self-efficacy, health goal attainment and self-management. Consistent with previous literature, self-management was described as an endpoint outcome resulting from identified health needs and strategies to cope with chronic disease on a daily basis [27]. Participants reported the meaningful role of knowledge acquisition in allowing for self-management to occur. The description of power over health differed from the concept of empowerment in the literature on chronic disease management, defining a process of active development and use of knowledge, skills, confidence, satisfaction and positive thinking to enable control over one’s life and self-health promotion [27]. The empowerment process, as it is defined in literature, was not expressed by patients and providers in our qualitative description. Rather, power over health was described as decisional authority over health acquired by the patient or transferred by the provider.

Physical health outcomes were described as improvements in physical symptoms as communicated by the participants, which included pain and physical symptom control, energy and weight management. Functional outcome was described differently in our study than in the concept analysis by Wang et al. [28]. The authors described the concept as measuring the level of activity required to perform daily tasks, while the participants in our analysis described this outcome as lowering the barriers to their autonomy in accomplishing daily tasks.

Psychological health was depicted as a reduction in general anxiety levels and an improvement in overall well-being. The literature describes psychological well-being as a positive mental state that helps people flourish [29]. Accordingly, participants in our study reported that accepting their health condition and feeling less guilty were key factors of psychosocial well-being.

Health-related behaviours outcomes were described as the introduction of new health behaviours to the patients’ routine or change and improvement in existing behaviours. Smoking habits and alcohol consumption were not considered by previous multimorbidity-adapted interventions as relevant outcomes [21]. Providers reported that while they were rare occurrences, some patients experienced improvements in these habits.

The study participants stated that improving their quality of life was the main general health outcome of the intervention. While not described by patients or providers in our interviews, the measurement of general self-rated health status is used in intervention studies for people with multimorbidity [21]. Patients also described the general health concept using other, similar wording such as a feeling of being healthy and a life-changing feeling. These two themes were used by patients to express their feelings about improvements in their general health.

Care satisfaction was described as a feeling of security and rightful follow-up. Two family physicians reported a decrease in unplanned use of physician services and emergency departments, but not by patients with multimorbidity. Care utilization has been used as an outcome in a previously published multimorbidity intervention paper [30] and has shown mixed reliability for primary care visits [31]. Further exploration of the care utilization outcome is needed to assess the feasibility of a patient report in the context of multimorbidity intervention.

This study highlights how the process of improvement differs from the general population. Mainly, the participants identified the awareness of the health status as a first step, the consciousness of the power that they can have over their health, the sense of responsibility over their treatment, the acceptance of their health status and the improvement over energy levels before improving physical activity.

### Strengths and limitations

This qualitative analysis provides a better understanding of the perspectives of both patients and providers on the results of an approach to care adapted to multimorbidity. The qualitative descriptive design uses the clinical experience of the research team throughout the data collection and analysis to ensure clinical relevance and application. This outcome description was developed using a triangulation of sources with both providers and patients offering real world descriptions of potential outcomes, and triangulation of interviewers’ and researchers’ backgrounds to enhance the validity of our data collection.

The first limitation is that the study looked at a single context of a particular chronic disease intervention for multimorbidity patients in which providers were trained in a multimorbidity-adapted care approach, which could have limited the range of outcomes identified and limits generalizability. A second limitation in the data collection method was the use of individual interviews only. Data collection including observation of clinical appointments or file reviews could have enhanced the description. However, this limitation is partly offset by the use of an iterative data collection process involving multidisciplinary interviewers and multiple sources (patients and healthcare providers).

### Implications for research and practice

Our analysis offers a stakeholder understanding of relevant outcomes that are observable by patients. This description could be of use for multimorbidity research design and intervention evaluation for policy and practice, to assess outcomes relevant to patients. This analysis offers insight into the way outcomes are observed and worded by stakeholders, which is highly relevant for the development of patient-reported outcome measures. The description of the improvement process can significantly impact the items needed and how they are worded to gather relevant information. A description from the stakeholders' perspective is a requirement by the ISOQOL for the validity of the development of a PROM that is relevant to and observable by patients [17]. This qualitative description in association with evidence from literature will be essential in ensuring content validity in the development process.

## Conclusions

Stakeholders described a wide range of outcome constructs that are relevant to and observable by patients and are in line with the clinical reality. The analysis provides a portrait of outcomes significant for the selection of clinical research outcome measures and the development of outcome measures.

## Data Availability

The datasets collected and analysed during the current study is available from the corresponding author on reasonable request.
